# Supporting Healthcare and Paramedic Employees (SHAPE)—study protocol for a single-blind, superiority randomised controlled trial evaluating cognitive therapy coaching for PTSD and MDD for health and social care workers in the United Kingdom

**DOI:** 10.1186/s13063-025-09341-4

**Published:** 2025-12-20

**Authors:** Jasmine Laing, Aimee McKinnon, Cathy Creswell, Anke Ehlers, Jennifer Wild

**Affiliations:** 1 Department of Experimental Psychology, University of Oxford, Oxford, UK; 2 Department of Psychiatry, University of Oxford, Oxford, UK; 3 Department of Psychiatry, Phoenix Australia Centre for Posttraumatic Mental Health, University of Melbourne, Melbourne, Australia

**Keywords:** Post-traumatic stress disorder, Major depressive disorder, Health and social care workers, Cognitive therapy for PTSD, Coaching intervention, Randomised controlled trial, Study protocol

## Abstract

**Background:**

This single-blind, superiority randomised controlled trial will evaluate the efficacy of cognitive therapy (CT) coaching for posttraumatic stress disorder (PTSD) and major depressive disorder (MDD) for health and social care workers in the UK compared to a waitlist condition.

**Methods:**

The trial will include two parallel conditions (intervention and waitlist) using a 1:1 allocation ratio. Assessors of treatment outcome will be blinded to trial condition. Ninety-two participants who meet criteria for PTSD or MDD will be randomly allocated to the intervention or waitlist condition, minimising for key clinical characteristics. Participants allocated to waitlist will be offered the SHAPE intervention afterwards. The intervention will be tailored CT coaching for PTSD and MDD called SHAPE. The primary assessment point is 8 weeks post-random allocation. Further assessment points are 4 weeks post-random allocation and 6 months after receiving the intervention to measure the long-term effects of SHAPE. The primary outcome is SCID-5 diagnosed PTSD and MDD at 8 weeks post-random allocation. The PTSD Checklist for DSM-5 (PCL-5) and the Patient Health Questionnaire (PHQ-9) will be used as secondary measures of PTSD and MDD to assess for symptom severity. Other secondary measures will assess for posttraumatic cognitions, responses to intrusive memories, resilience, wellbeing, and symptom severity for generalised anxiety disorder and insomnia. Health economic analyses will compare the two conditions on quality of life at 8 weeks post-random allocation and compare productivity loss, resource use, and health problems at pre- and post-intervention for everyone who received SHAPE. Patient and provider experience of the SHAPE intervention will be assessed by semi-structured interviews at post-intervention.

**Discussion:**

This trial will be the first to evaluate the efficacy of CT coaching for PTSD and MDD for UK health and social care workers compared to a waitlist control. Since PTSD symptoms can improve naturally over time, comparison to a waitlist condition allows intervention effects to be distinguished from the effects of time alone. Findings from this trial will inform adaptations that may make the intervention more precise and effective.

**Trial registration:**

ISRCTN57105098. Registered prospectively on February 21, 2023.

**Supplementary Information:**

The online version contains supplementary material available at 10.1186/s13063-025-09341-4.

## Administrative information

Note: the numbers in curly brackets in this protocol refer to SPIRIT 2025 checklist item numbers. The order of the items has been modified to group similar items (see https://www.equator-network.org/reporting-guidelines/spirit-2013-statement-defining-standard-protocol-items-for-clinical-trials/).

**Table:** 

Title {1a}	Supporting Healthcare and Paramedic Employees (SHAPE)—study protocol for a single-blind, superiority randomised controlled trial evaluating cognitive therapy coaching for PTSD and MDD for health and social care workers in the United Kingdom
Structured summary {1b}	Trial registration: ISRCTN57105098. Registered prospectively on February 21, 2023 Contact for public and scientific queries: Professor Jennifer Wild jennifer.wild@psy.ox.ac.uk Public title: Supporting Healthcare and Paramedic Employees (SHAPE) with Cognitive Therapy Coaching for PTSD and Depression—protocol for an RCT Scientific title: Supporting Healthcare and Paramedic Employees (SHAPE)—study protocol for a single-blind, superiority randomised controlled trial evaluating cognitive therapy coaching for PTSD and MDD for health and social care workers in the United Kingdom Countries of recruitment: UK (England, Scotland, and Wales) Health conditions studied: PTSD and major depressive disorder (MDD) Intervention: A tailored cognitive therapy coaching intervention for PTSD and MDD called SHAPE Study type: Single-blind, superiority randomised controlled trial that will include two parallel conditions (intervention and waitlist) using a 1:1 allocation ratio. Assessors of treatment outcome will be blinded to trial condition. Ninety-two participants who meet criteria for PTSD or MDD will be randomly allocated to the intervention or waitlist condition, minimising for key clinical characteristics. Date of first enrolment: February 24, 2023 Recruitment status: Complete, participants are no longer being recruited or enrolled. Primary outcome: Absence of PTSD and MDD using the Structured Clinical Interview for the DSM-5 at 8 weeks post-intervention allocation Ethics review: approved on December 7, 2022, by the Medical Sciences Interdivisional Research Ethics Committee at the University of Oxford, ref R80469/RE008
Protocol version {2}	Version 1.3, October 20, 2025
Names, affiliations, and roles of protocol contributors {3a}	Jasmine Laing^a^, Aimee McKinnon^a^, Cathy Creswell^a,b^, Anke Ehlers^a^, Jennifer Wild^a,c*^ ^a^Department of Experimental Psychology, University of Oxford, UK ^b^Department of Psychiatry, University of Oxford, UK ^c^Phoenix Australia Centre for Posttraumatic Mental Health, Department of Psychiatry, University of Melbourne, Australia *Corresponding author and PI JW and AE designed the intervention. JW and JL planned the trial design. JL drafted the protocol. JL and JW drafted the statistical analysis plan with consultation from two senior statisticians from the NIHR Applied Research Council for Oxford and Thames Valley and the National Institute for Health and Care Research (NIHR) Oxford Health Biomedical Research Centre. JW drafted the health economics plan with consultation from a senior health economist from the NIHR Applied Research Council for Oxford and Thames Valley. CC has provided continuous input into the trial design and planning. JL is coordinating the trial. JW and AM provide clinical supervision. All authors contributed to the refinement and revisions of the study protocol and approved the final manuscript.
Name and contact information for the trial sponsor {3b}	Jennifer Wild (principal investigator), Department of Experimental Psychology, University of Oxford, UK (jennifer.wild@psy.ox.ac.uk)
Role of sponsor and funders in design, conduct, analysis, and reporting of trial; including any authority over these activities {3c}	This is an investigator-initiated randomised controlled trial. The funders and sponsor played no role in the design of the study, data collection, analysis, interpretation of data, and writing of the manuscript.
Composition of the coordinating centre and trial steering committee {3d}	The coordinating centre for this trial is the Department of Experimental Psychology at the University of Oxford. The Trial Steering Committee (TSC) consists of members independent to the University of Oxford and who are not directly involved in the running of the SHAPE trial. The composition of the TSC includes an independent chair, two members with scientific and academic background in the field, a PPIE representative, a biostatistician, and a representative of the funding organisation who has extensive experience in implementation and translation of interventions. The principal investigator and members of the main research team will be invited to join TSC meetings as observers. Five out of the six full members of the TSC do not have any relationship with the trial investigators, funder(s), or sponsor that would allow them to be influenced by these bodies. One full member of the TSC is connected to the main funder of the trial, however, will not be involved in any of the major decisions. The TSC are responsible for overseeing the trial and assessing its progress and adherence to the trial protocol and data analysis plan, overseeing recruitment rates and completeness of data collection, assessing patient safety, and providing advice on major decisions to do with trial continuation and risk and safeguarding matters. The TSC will meet once per year to discuss the progress of the trial.

## Open science

**Table:** 

Trial registration {4}	ISRCTN registry, ISRCTN57105098, February 21, 2023, https://www.isrctn.com/ISRCTN57105098
Protocol and statistical analysis plan {5}	The results of this trial will be published in peer-reviewed scientific journals with open access. All participants in this trial have given permission for their aggregated anonymised data to be shared with a data repository, such as Ox-data or the UK data archive. Some participants in the trial have consented for their raw research data to be shared with other researchers, including those working outside of the UK and the EU, for use in other research. These data will be available upon request from Prof. Jennifer Wild (jennifer.wild@psy.ox.ac.uk). Raw data will be anonymised, and participants will not be identifiable. The raw data will become available after the publication of the results of the trial so that they can be used for meta-analyses or specified additional analyses. Only numerical raw data from the self-report questionnaires and SCID-5 assessments will be made available to other researchers. Audio recordings from the SCID-5 assessments and the qualitative interviews with participants will not be made available and will be destroyed by the end of the research study. The protocol and statistical analysis plan will be made publicly available for researchers to access. The protocol pre-print can be accessed on Research Square here: https://www.researchsquare.com/article/rs-5369299/v1 The statistical analysis plan can be accessed from Open Science Framework here: https://osf.io/ydsre
Data sharing {6}	Trial materials can be obtained from the first author (JL). Participants in this study have given permission for their aggregated anonymised data to be shared with a data repository, such as Ox-data or the UK data archive. For participants who consent to their raw research data to be given to other researchers, including those working outside of the UK and the EU, to be used in other research studies, this will be available upon request from Prof Jennifer Wild (jennifer.wild@psy.ox.ac.uk).
Funding {7a}	NIHR Applied Research Collaboration for Oxford and Thames Valley University of Oxford COVID Research Response Fund Wellcome Trust, grant 00070 awarded to JW National Institute for Health and Care Research (NIHR) Oxford Health Biomedical Research Centre This research is funded and supported by the National Institute for Health and Care Research (NIHR) Applied Research Collaboration Oxford and Thames Valley, at Oxford Health NHS Foundation Trust. JL and CC receive funds from the NIHR Applied Research Collaboration Oxford and Thames Valley at Oxford Health NHS Foundation Trust. Development of the SHAPE intervention was initially funded by the University of Oxford COVID Research Response Fund and the Wellcome Trust, grant 00070, awarded to JW. Additional financial support for the trial has come from the National Institute for Health and Care Research (NIHR) Oxford Health Biomedical Research Centre (BRC) (CC and AE). The views expressed are those of the author(s) and not necessarily those of the NHS, the NIHR, the Wellcome Trust, or the Department of Health and Social Care.
Conflicts of interest {7b}	The authors declare that they have no competing interests.
Dissemination policy {8}	The results of the trial will be published in peer-reviewed international journals. All publications for this study will be made available open access. Information about the results will be made available to the participating services and participants if requested. All members of the research team, coaches, assessors, and stakeholders who participate in the study and provide intellectual input to the trial design, execution, statistical analyses, or write-up will be acknowledged in publications. Model consent forms and additional study material can be made available upon request.

## Introduction

### Background and rationale {9a}

Posttraumatic stress disorder (PTSD) is a debilitating stress disorder that some people develop after experiencing, witnessing, or repeatedly hearing about a life-threatening or dangerous event. Individuals with PTSD typically re-experience the trauma in the form of distressing memories, nightmares, or flashbacks. They may avoid trauma triggers, such as people, places, or conversations that reactivate memories of the traumatic experience. They may also perceive themselves more negatively and experience a range of hyperarousal symptoms, such as sleep problems, difficulties concentrating, and feeling on edge. Whilst PTSD is the more common outcome following trauma, major depression can also develop, without co-occurring PTSD or as a disorder secondary to PTSD [1, 2]. Although many individuals experiencing PTSD and major depression will receive some form of psychological treatment, some individuals with PTSD and/or major depression may go on to recover without treatment [3, 4].

Health and social care workers (HSCWs) are at risk of developing PTSD and major depressive disorder (MDD) due to exposure to trauma at and outside of work or in their childhood [5–9]. Limited evidence-based interventions are available that are tailored to their needs, such as providing flexibility to be accessed out of hours, during breaks, or remotely [10–13]. Improving psychological wellbeing by facilitating recovery from mental health disorders may benefit HSCWs and the patients they treat [14, 15] and may reduce the exodus of qualified workers from the NHS [16].

This randomised controlled trial aims to evaluate a tailored cognitive therapy (CT) coaching intervention for PTSD and MDD for HSCWs in the UK. The tailored CT intervention *Supporting Healthcare and Paramedic Employees* (SHAPE) was designed during the COVID-19 pandemic to treat healthcare workers with PTSD and MDD (Wild J, McKinnon A, Wilkins A, Storch C, Browne H, & Ehlers A. Cognitive therapy coaching for PTSD and depression symptoms in healthcare workers repeatedly exposed to trauma: a pilot evaluation of SHAPE, under review). The intervention is brief, flexible, and delivered remotely, making it more accessible for HSCWs’ busy, unpredictable, and fluctuating schedules. SHAPE draws on the treatment targets of cognitive therapy for PTSD (CT-PTSD) [17], rumination-focused cognitive behavioural therapy [18], and the evidence base for cognitive and behavioural risk factors for PTSD and MDD in paramedics [2, 19]. For a detailed description of SHAPE, please see Wild J, McKinnon A, Wilkins A, Storch C, Browne H, & Ehlers A. Cognitive therapy coaching for PTSD and depression symptoms in healthcare workers repeatedly exposed to trauma: a pilot evaluation of SHAPE, under review).

#### Explanation for choice of comparator {9b}

The comparator will be an 8-week waitlist control condition. Since some individuals with PTSD experience recovery over time, a waitlist comparison allows us to distinguish intervention-specific effects from those associated with the passage of time.

### Objectives {10}

The primary objective of this trial is to determine whether the SHAPE intervention is superior to a waitlist condition in reducing rates of PTSD and MDD in UK HSCWs 8 weeks after randomisation.

Secondary objectives of the trial are to determine whether the SHAPE intervention leads to greater improvements in symptoms of PTSD, MDD, generalised anxiety disorder (GAD), complex PTSD (C-PTSD), and insomnia, as well as greater improvements in wellbeing, resilience, and quality of life compared to a waitlist condition 8 weeks after treatment allocation. We will also explore the stability of treatment effects by assessing the same measures 6 months after the end of the intervention, as well as explore potential correlates of treatment outcome.

Additionally, we aim to measure productivity loss, health problems, and health service use before and 6 months after having the SHAPE intervention in a health economics evaluation and determine whether SHAPE is associated with greater improvements in quality of life compared to waitlist.

The trial will also investigate the acceptability, accessibility, and suitability of the SHAPE intervention for HSCWs receiving SHAPE and the coaches delivering SHAPE through semi-structured interviews. We will employ the framework analysis method to analyse the findings both inductively and deductively.

#### Hypotheses

##### Primary outcome

We predict that the SHAPE intervention will achieve a significantly higher recovery rate than the waitlist condition. Specifically, we predict that the intervention condition will achieve a recovery rate of at least 50%, compared to an estimated 16% recovery rate in the waitlist condition, 8 weeks after randomisation. The recovery rate for the intervention was informed by the NHS Talking Therapies (formerly known as Improving Access to Psychological Therapies, IAPT) treatment recovery target of 50% and through discussions with service leads working in NHS wellbeing hubs about the minimum recovery rate they would find acceptable for patients receiving psychological treatment. It was also supported by a previous pilot study conducted on SHAPE during the pandemic (Wild J, McKinnon A, Wilkins A, Storch C, Browne H, & Ehlers A. Cognitive therapy coaching for PTSD and depression symptoms in healthcare workers repeatedly exposed to trauma: a pilot evaluation of SHAPE, under review).

##### Secondary outcomes

We predict that the SHAPE intervention will be superior to the waitlist condition by achieving significantly greater change in clinical symptoms (i.e. PTSD, MDD, GAD, C-PTSD, and insomnia) at 8 weeks post-treatment allocation. We also predict that the SHAPE intervention will be superior to the waitlist condition by achieving significantly greater change in wellbeing, resilience, and quality of life measures at 8 weeks post-treatment allocation. For the correlates of change analysis, we hypothesise that changes in cognitive processes (posttraumatic cognitions, maladaptive responses to intrusive memories) from baseline to mid-intervention (week 4) will predict the extent of PTSD and MDD symptom reduction (week 8), controlling for baseline scores. We hypothesise that changes in cognitive processes (posttraumatic cognitions, maladaptive responses to intrusive memories) from baseline to week 8 will correlate with PTSD and MDD symptom reduction from baseline to post-intervention (week 8), controlling for baseline scores.

##### Long-term follow-up outcomes

We predict that the effects of the SHAPE intervention will be maintained at the 6-month follow-up for the primary and secondary outcome measures.

## Methods: patient and public involvement, trial design

### Patient and public involvement {11}

Involvement of, and contributions from, patients and the public took place during the design phase of this trial. Service leads working within NHS staff wellbeing hubs were consulted when determining the power calculation for the trial in regard to what they deemed would be an acceptable recovery rate for a psychological intervention. Additionally, meetings were held with participants who had received SHAPE as part of a pilot study to inform interview guides for the complementary qualitative study. Plans to involve patients and the public in the reporting and dissemination of findings from the trial and the complementary qualitative study are also in place.

### Trial design {12}

The trial is a randomised controlled superiority single-blind trial with two parallel conditions using a 1:1 allocation ratio. Participants and coaches will not be blind to participants’ condition allocation due to the nature of the interventions. Assessors collecting primary and secondary outcome measures at 8 weeks post-randomisation and 6 months post-intervention follow-up will be blinded to the trial arm.

## Methods: participants, interventions, and outcomes

### Trial setting {13}

The study will be conducted with participants across the UK. The study will be conducted over the phone and online (Microsoft Teams). The project’s administrative hub will be located at the central site, the Department of Experimental Psychology, at the University of Oxford, UK. Most research team members will be located at the central site. Given the remote nature of the trial and the intervention, some assessors conducting the clinical assessments, coaches delivering the intervention, and one member of the research team will be based outside of the central site. Members located outside of the central site will perform study operations remotely, in a private, secure space, typically from home, and will be regularly contacted and monitored by the trial manager (JL) based at the central site. Participants will be recruited remotely using the study team’s networks, social media, snowball sampling, and through independent organisations offering occupational and mental health support to HSCWs.

### Eligibility criteria for participants {14a}

Table 1 outlines the inclusion and exclusion criteria for the study. Eligibility criteria were chosen to allow for a wide range of HSCWs experiencing symptoms of PTSD or MDD to participate in the trial.

**Table 1:** Randomised controlled trial for Supporting Healthcare and Paramedic Employees (SHAPE): Inclusion and exclusion criteria

Inclusion criteria
1. Aged 18 years and older
2. Patient-facing health or social care worker—professions can include nurse, doctor, paramedic, midwife, care worker, ambulance service team member, allied health professional, mental health professional, healthcare support worker, medical associate, pharmacist, care assistant, and nursing or medical student working in a healthcare setting
3. PTSD or MDD is the primary disorder requiring treatment (i.e. the main clinical condition currently warranting treatment, as determined during the clinical assessment)
4. Diagnosed with PTSD or MDD on the Structured Clinical Interview for DSM-5 (SCID-5) PTSD or MDD module
5. Willing and able to provide informed consent
6. Willing to be randomised
7. If taking psychotropic medication, the dose must be stable for at least 1 month before randomisation and remain stable throughout the trial
8. If currently receiving psychological therapy, the treatment must have ended before randomisation
9. Any current re-experiencing symptoms are linked to no more than 2 discrete traumatic events that have occurred at or outside of work, or several traumatic episodes during a longer period of high threat (e.g. domestic abuse, childhood abuse, COVID-19, prolonged work trauma)
Exclusion criteria
1. Imminent, persistent, clinically significant risk which needs immediate attention, or which would interfere with the capacity to engage in the trial
2. Request for face-to-face treatment for PTSD or MDD
3. Preference for a counselling approach, or trauma-focused therapy within NHS Talking Therapies
4. Previously received the SHAPE intervention before joining the trial

**Fig. 1:**
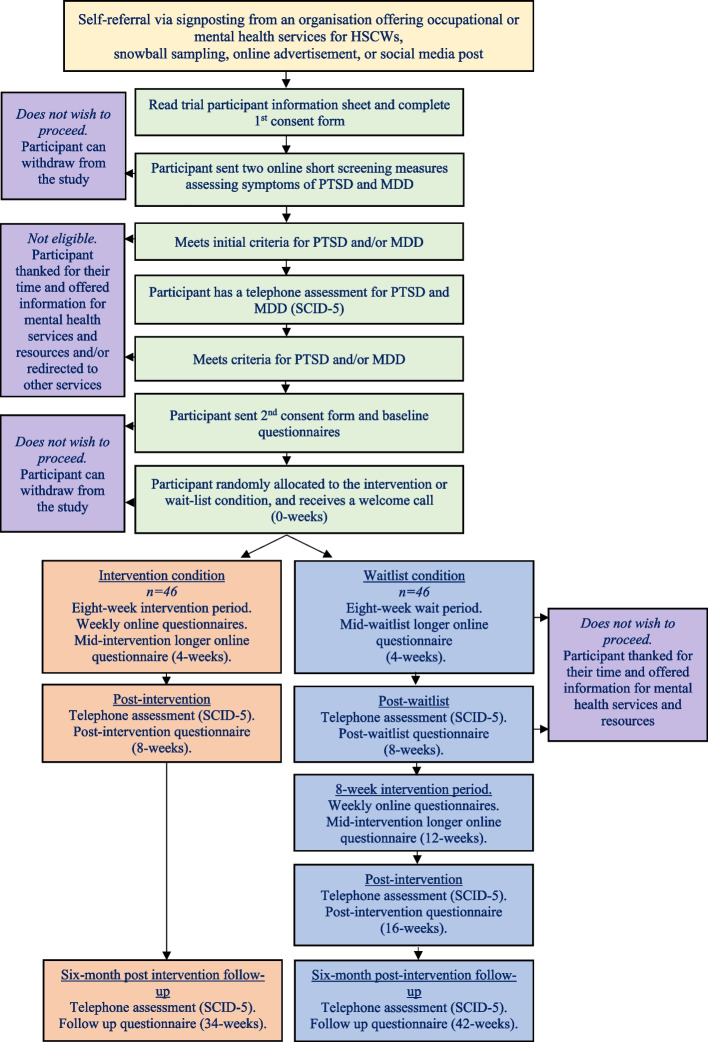
Stages of the SHAPE randomised controlled trial for participants

A flow chart showing the different stages of enrolment and recruitment into the trial is illustrated in Fig. 1. Potential participants will be directed to the trial information sheet and first consent form, which seeks consent to be assessed. The information sheet contains information on the study aims, allocation process, study design, potential risks and benefits of taking part, reimbursement for taking part, confidentiality, data sharing and protection, ethics, freedom to withdraw, and contact details of the study team. Participants will also have the option to ask a question by clicking a button that will notify the study team to get in contact with them. After a participant consents to being assessed, they will be sent two short screening measures that assess symptoms of PTSD (Posttraumatic Stress Disorder Checklist for DSM-5; PCL-5) [20] and MDD (Patient Health Questionnaire; PHQ-9) [21]. If a participant scores above the study’s specified cut-off points for either screening measure (10 on the PHQ-9 or 20 on the PCL-5) and satisfies eligibility criteria, the trial lead (JL) will contact the participant to schedule a telephone clinical assessment to confirm eligibility. A score of 20 or more on the PCL-5 was chosen since a recent study with UK healthcare workers demonstrated that the clinical cut-off score of 31 tended to underdiagnose participants with PTSD [5]. Telephone assessments will be conducted by trained assessors who will complete the Structured Clinical Interview for DSM-5 for PTSD and MDD with the participant. If the telephone assessment confirms that the participant is eligible for the trial (they are diagnosed with either PTSD or MDD and satisfy study criteria), the participant will be invited to complete a second consent form, which establishes consent to take part in the trial. Once a participant completes the second consent form, they will be sent a set of baseline questionnaires to complete. After a participant completes the baseline questionnaires, they will be randomised into the intervention or waitlist condition. The participant will be assigned a coach, and they will receive a short welcome call with the trial lead to explain the next steps of the trial and answer any remaining questions. Participants who are not eligible for the trial or who do not wish to participate in the trial will be thanked for their time and will be signposted to other services if relevant. They will also receive a £10 e-voucher as an honorarium for taking part in the telephone assessment.

### Eligibility criteria for individuals who will deliver the intervention {14b}

Coaches delivering the SHAPE intervention require: (1) a psychology undergraduate degree or higher, or (2) current employment in a role related to psychological therapies (e.g. psychology research assistant, psychological wellbeing practitioner, CBT therapist, clinical psychologist). In addition, coaches must have attended a 1-day intensive SHAPE training workshop, have treated two training cases with SHAPE, and be available to take part in weekly supervision sessions with a trial clinical supervisor.

## Intervention and comparator

### Intervention and comparator description {15a}

#### SHAPE condition

After screening, assessment, and randomisation, participants randomly allocated to the intervention condition will receive the SHAPE intervention over a period of 8 weeks. SHAPE will be delivered remotely over the telephone or via audio link through Microsoft Teams, dependent on participant preference to further enhance the accessibility of SHAPE coaching and reduce potential barriers (e.g. limited access to the internet or to an electronic device, difficulties with computer literacy). Participants will receive approximately 6 coaching calls (1 call per week) with the expectation that participants or coaches may need to skip a week (due to sickness, annual leave, holidays, etc.). In circumstances where additional clinical support is warranted and would be in the participant’s clinical best interests, up to 2 more sessions can be offered (e.g. prolonged exposure to multiple traumatic events, new trauma that emerges during treatment, an ongoing court case). Coaching calls will last between 30 and 50 min and will focus on a specific tool (as outlined in Table 2).

**Table 2:** Tools typically used in SHAPE weekly coaching calls

Core tools
1. THEN vs. NOW for re-experiencing symptoms
2. IF THEN plans for rumination
3. Planning ahead for low mood and avoidance
4. Surveys and behavioural experiments for updating unhelpful thoughts
5. Imagery to create a sense of continuity in the present with what has been lost in the past
6. Responsibility pie charts for guilt and self-blame
7. Realistic risk and worry diary for worry
8. Blueprint for beating stress and low mood

During supervision, coaches will plan their future coaching calls with their designated supervisor, who will be a clinical psychologist on the SHAPE team. Coaches and supervisors will discuss which tools would be most useful to cover in each call to address the key symptoms endorsed on the weekly PTSD and MDD symptom measures. Table 2 provides a list of the core tools typically used in the SHAPE intervention and the symptoms they target.

Participants will have the option to complete a supplementary online learning module consolidating a skill covered in the coaching call for home practice. There are seven modules that participants can access depending on their presenting symptoms. Table 3 highlights the seven online modules used in SHAPE. Modules take between 15 and 30 min to complete and can be accessed on a laptop, computer, tablet, or phone. If a participant is interested in completing the module, they will be sent a link from their coach via email after they have had their weekly call. The link will not expire, meaning participants will have unlimited access to the module and can retake the module whenever they need, even after the trial ends. The online modules have been designed for SHAPE and include examples that are relevant to the healthcare context.

**Table 3:** SHAPE online learning modules

Online module
1. Dealing with Unwanted Memories: Then vs Now
2. Habits & Dwelling—How to Change Them
3. Planning Ahead
4. Get out of Your Head with Helpful Thinking
5. Helpful and Unhelpful Attention—It Matters what you Focus On
6. Dealing with Guilt and Self-blame
7. Transforming Worry and Improving Performance

#### Waitlist condition

Participants randomly allocated to the waitlist condition will not receive any therapeutic support from their assigned coach or the research team during the 8-week waiting period. Participants are asked not to take up psychological support during the waiting period (i.e. CBT, counselling) or change their current psychotropic medication if they are already taking it to allow for a clean comparison between conditions. If a participant wishes to access the SHAPE intervention, they will be able to receive the intervention after they complete their post-wait measures and have their second telephone assessment at 8 weeks post-random allocation. If a participant no longer wishes to receive the intervention, they can opt out and will be provided with relevant information on alternative services if requested.

### Criteria for discontinuing or modifying allocated intervention/comparator for a trial participant {15b}

We deem our trial to be low risk due to the nature of the intervention and the trial design. However, thoughts of death, suicidal ideation, and increased risky behaviours can be symptoms of MDD and PTSD. Risk will be carefully monitored throughout the trial through the questionnaires, the clinician-administered assessments, and from regular weekly contact with the coach for participants assigned to the intervention condition. All participants will be made aware of the study team’s contact details in the study information sheet should they need to be in touch regarding risk. A risk and safeguarding plan has been developed for the trial, and all coaches and assessors have been briefed on how to use it and the steps to take if a participant demonstrates signs of risk or if a safeguarding concern emerges.

#### Participants in the intervention condition

Some participants in the 8-week intervention condition might experience worsening of symptoms during this time. For some participants, an unexpected life event might occur whilst they are in the intervention condition. If this event causes severe distress and risk emerges, the study team will assess if a more urgent support service is required. If the study team deems that a participant would require a more urgent support service, the participant will be withdrawn from the study and referred to the relevant support service, with consent. If a participant needs to take some time off due to the unexpected life event, their intervention will be paused, and they will be able to resume coaching within 6 months after pausing or have the option to withdraw.

#### Participants in the waitlist condition

Some participants in the 8-week waitlist condition might experience worsening of symptoms during this time due to the progression of symptoms or factors such as an unexpected life event. The study team will be able to detect this in the participant’s 4-week and 8-week questionnaires. The study team will contact the participant and conduct a risk assessment if they detect an increase in risk from baseline. If it is deemed that the participant would benefit from immediate assessment or treatment from a mental health service, then the study team will withdraw the participant from the trial and refer them to the relevant support service.

### Strategies to improve adherence to intervention/comparator protocols {15c}

#### For participants

Participants will be regularly contacted by members of the research team to ensure they are completing measures and attending calls. All participants will receive a welcome call after being randomised to ensure they understand the next steps of the trial and to allow for any questions to be answered. Participants will be compensated with an electronic voucher for each assessment they complete. For participants who are eligible to take part in the trial, they will receive their e-vouchers after completing their second assessment 8 weeks post-random allocation to reduce attrition and improve data completeness.

#### For coaches

Coaches will receive weekly online individual or group supervision to facilitate fidelity to the SHAPE intervention. All coaches will have completed a 1-day SHAPE training course and have completed two training cases under close supervision. Coaches will have access to a SHAPE information pack that contains information on the SHAPE treatment tools, worksheets for each tool to give to participants, trial inclusion and exclusion criteria, the trial procedure, the safeguarding protocol, and answers to frequently asked questions. Coaches will also have access to the online modules that accompany the SHAPE intervention tools that they can use to refresh their learning and understanding of the core tools used in SHAPE. Finally, coaches will complete a coach log after each call which asks for the duration of the call, the tools covered in the call, and the duration of administration and planning involved for each call to evaluate compliance and length of time.

#### For assessors

All assessors received training to conduct the SCID-5 for PTSD and MDD by the trial lead or lead clinical psychologist. All assessors administering the SCID-5 telephone assessment will have a script to follow before and after administering the two standardised SCID-5 modules to allow for telephone assessments to be consistent and standardised and to reduce possible assessment effects. All telephone assessments will be recorded, and 10% of the assessments will be double coded to assess for inter-rater reliability. In addition, the first five telephone assessments conducted by each assessor will be double coded by the lead researcher to ensure accuracy and consistency in their initial assessments. Assessors need to accurately diagnose PTSD and MDD in five consecutive calls in order to continue to conduct blind assessments during the trial. A total of 10% of remaining assessments will be chosen at random over the course of the trial.

If the lead researcher is unsure about a diagnosis made by an assessor, they will consult with one of the lead clinical psychologists on the trial who will listen to the recording to confirm the final diagnosis. For assessments where a participant accidentally discloses information revealing what condition they were assigned to, this information will be removed from the recording, and the assessment will be re-rated by another blind assessor.

#### For the research team

Only the lead researcher for the trial will conduct the welcome call and will follow a welcome call script to ensure that all welcome calls are standardised to reduce potential therapeutic effects before the intervention or waitlist condition begins.

### Concomitant care permitted or prohibited during the trial {15d}

Prior to beginning treatment, all participants will be instructed to refrain from receiving other psychological treatment during the trial. Participants will be advised that trial eligibility requires that they do not start new psychotropic medications during the trial and remain on a stable dose of their current psychotropic medication if they are taking medication. All urgent and unexpected concomitant psychological interventions, treatments, and changes in medication that do occur during the trial will be documented in the research trial records as soon as the research team are made aware of this.

### Outcomes {16}

All outcomes will be measured and compared between each treatment condition, unless otherwise specified.

#### Primary outcome

- Absence or presence of PTSD and MDD at 8 weeks post-random allocation, assessed with the Structured Clinical Interview for DSM-5 (SCID-5; PTSD and MDD modules) [22].

#### Secondary outcomes

- PTSD symptom severity at 4 and 8 weeks post-random allocation and at 6 months post-intervention completion, measured with the PCL-5 [20].
- MDD symptom severity at 4 and 8 weeks post-random allocation and at 6 months post-intervention completion, measured with the PHQ-9 [21].
- GAD symptom severity measured at 4 and 8 weeks post-random allocation and at 6 months post-intervention completion with the Generalised Anxiety Disorder Scale (GAD-7) [28].
- Insomnia symptom severity measured at 8 weeks post-random allocation with the Insomnia Sleep Index (ISI) [25].
- C-PTSD symptom severity measured at 8 weeks post-random allocation and at 6 months post-intervention completion with the International Trauma Questionnaire (ITQ) [27].

#### Process measures

- Frequency of maladaptive responses to intrusions measured at 4 and 8 weeks post-random allocation and at 6 months post-intervention completion with the Responses to Intrusions Questionnaire Short Version (RIQ-s) [29].
- Posttraumatic cognitions measured at 4 and 8 weeks post-random allocation and at 6 months post-intervention completion with the Posttraumatic Cognitions Inventory Short Version (PTCI-s) [31].

#### Wellbeing and resilience measures

- Wellbeing measured at 8 weeks post-random allocation and at 6 months post-intervention completion with the Warwick-Edinburgh Mental Wellbeing Scale (WEMWBS) [32].
- Resilience measured at 8 weeks post-random allocation and at 6 months post-intervention completion with the Connor-Davidson Resilience Scale 25 (CD-RISC-25) [34].
- Social support from family/friends and work/colleagues at 8 weeks post-random allocation and at 6 months post-intervention completion measured with the social support scales adapted from Sarason et al. [35].
- Life and job satisfaction questions at 4 and 8 weeks post-random allocation and at 6 months post-intervention completion.

#### Health economic measures

This study will also collect health economics outcomes to assess resource utilisation, productivity loss, quality of life, and health status. Health economic measures will be administered at baseline, 8 weeks post-random allocation, and 6 months post-intervention completion for secondary analyses.

#### Qualitative feedback

Semi-structured interviews exploring participants’ and coaches’ experiences of the SHAPE intervention. Constructive feedback will be collected from participants and coaches about the perceived acceptability, accessibility, and suitability of SHAPE to guide future improvements to the intervention. Semi-structured interviews will take place shortly after participants complete their post-intervention telephone assessment to allow feedback to be derived that is minimally affected by the passage of time. Interviews with wellbeing coaches will take place during the period that coaches are delivering the SHAPE intervention, or shortly afterward.

### Adverse event reporting and harms {17}

Participant risk and safety will be monitored throughout the trial. A protocol for defining and recording serious adverse events (SAEs) and adverse events (AEs) has been developed for the trial that will be referred to throughout the trial (see Appendix 1). If a SAE or AE is reported from a participant, an AE or SAE report form (as appropriate) will be filled out by the safeguarding lead who is on-call and also sent to the PI to complete and review. Only SAEs will be reported to the TSC, unless an AE is recurring or has been reported more than once for a participant.

For participants assigned to the intervention condition, SAEs and AEs will be monitored through screening questionnaires, SCID-5 telephone assessments, weekly questionnaires, and during coaching calls. For participants assigned to the waitlist condition, SAEs and AEs will be monitored with the study questionnaires and SCID-5 telephone assessments at baseline, 4-week post-random allocation, and 8-week post-random allocation.

During SCID-5 telephone assessments, assessors will be asked to complete a brief risk assessment if a participant discloses suicidal ideation. After the assessment, assessors will be required to consult with the available safeguarding lead on the research team for guidance on what necessary action needs to be taken, and the PI will be informed. A SHAPE AE or SAE report form will be completed by the safeguarding lead (see Appendix 2) and PI (see Appendix 3) and safely stored in a secure online location on Nexus 365 OneDrive for Business.

During calls, coaches will be asked to notify the assigned safeguarding lead directly should they be concerned at any time that a participant has caused, or is likely to cause, significant harm to themselves or others. Coaches will conduct risk assessments if they deem a participant to be showing signs of risk during the call, or if item 9 on the PHQ-9 increases from their baseline value. If a participant is deemed to be at high risk, the study team will inform the participant’s GP with consent from the participant in order to ensure participant safety. If a coach is concerned that a participant is believed to likely cause imminent significant harm to others, they will need to pass this information onto the assigned safeguarding lead, who will be required to inform the appropriate authorities directly.

All SAEs and AEs will be reported in a reporting form (see Appendix 2 and 3) and logged in a risk and safeguarding report log that will be stored in a secure online location on Nexus 365 OneDrive for Business for the study team to access. Only the main research team members will be able to access the specific AE and SAE reports. The number and type of SAEs will be reported in the main trial publication.

### Participant timeline {18}

**Fig. 2:**
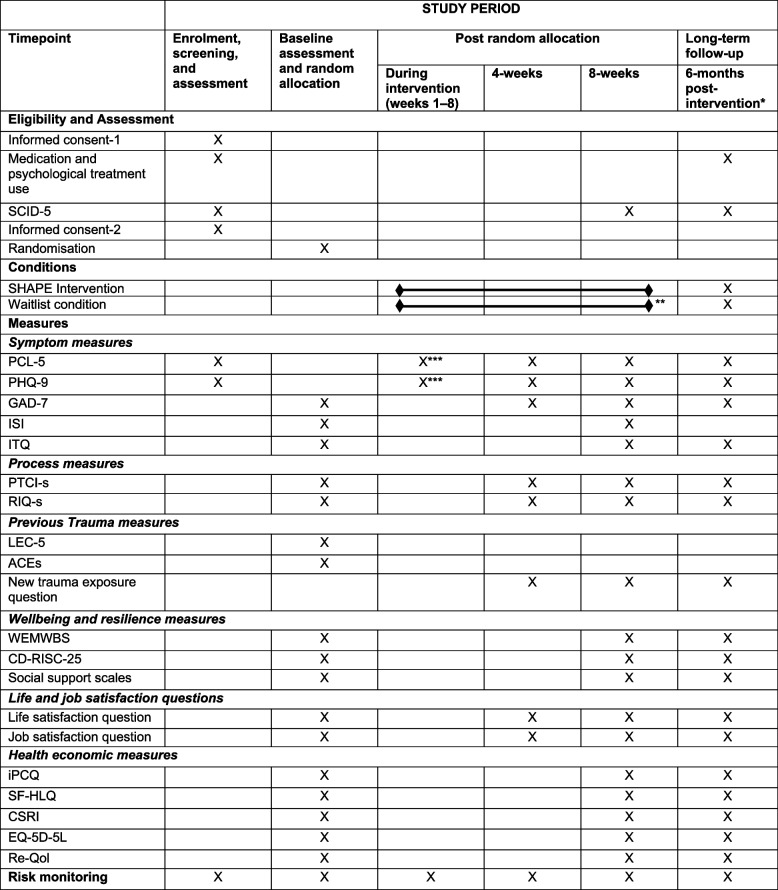
Schedule for forms, measures, interventions, and assessments in the SHAPE randomised controlled trial. *6 months post-intervention for participants randomly allocated to intervention or to waitlist who then receive the intervention, or 6 months post-waitlist for participants who complete the waitlist and who do not take up the intervention. **Followed by SHAPE intervention for participants who complete the waitlist condition and who would like to receive SHAPE coaching. ***Every week participants in SHAPE coaching receive a short weekly questionnaire (i.e. PCL-5 and PHQ-9) except in week 4 when they are sent the mid-intervention questionnaire. Informed consent-1 = Informed consent for eligibility assessment; *PCL-5* = PTSD Checklist for DSM-5; *PHQ-9* = Patient Health Questionnaire; *SCID-5* = Structured Clinical Interview for DSM-5 (PTSD and MDD modules); Informed consent-2 = Informed consent for assessment/trial; *GAD-7* = Generalised Anxiety Disorder Scale; *ISI* = Insomnia Sleep Index; *PTCI-s* = Posttraumatic Cognitions Inventory short version; *RIQ-s* = Responses to Intrusions Questionnaire short version; *LEC-5* = Life Events Checklist for DSM-5; *ITQ* = International Trauma Questionnaire; *ACE-Q* = Adverse Childhood Experiences Revised Questionnaire; *WEMWBS* = Warwick-Edinburgh Mental Wellbeing Scale; *CD-RISC-25* = Connor-Davidson Resilience Scale 25; *iPCQ* = iMTA Productivity Cost Questionnaire; *SF-HLQ* = Short Form Health and Labour Questionnaire; *CSRI* = Client Service Receipt Inventory; *EQ-5D-5L* = EuroQol-5 Dimensions-5 Levels health survey; *Re-QoL* = Recovering Quality of Life Questionnaire

A summary and timeline of the administration time points for forms, measures, interventions, and assessments administered in the RCT is shown in Fig. 2.

#### SHAPE condition timeline

Participants receiving the SHAPE coaching will be sent the PCL-5 and PHQ-9 at the end of each week to assess for changes in symptoms of PTSD and MDD respectively and to help in planning the upcoming coaching call. These measures will be sent via a secure online link for participants to complete on a digital device. Participants will need to enter their unique ID number before completing the self-report measures to allow the research team to link IDs to responses. Halfway through the intervention, participants will receive a longer set of measures to complete. Eight weeks after randomisation, participants will complete a longer set of post-intervention questionnaires and have another SCID-5 telephone assessment to re-assess for PTSD and MDD. Six months after receiving the SHAPE intervention, a final set of follow-up questionnaires will be sent, and participants will have a final remote SCID-5 telephone assessment to re-assess for PTSD and MDD. All post-randomisation SCID-5 assessments will be conducted by blinded assessors, unaware of the participant's allocation.

#### Waitlist condition timeline

Participants allocated to the waitlist condition will be sent a set of questionnaires halfway through the waitlist period to assess for symptom change and risk. If risk is detected, a member of the research team will contact the participant to assess risk and signpost if necessary. At the end of the waiting period, participants will receive another set of questionnaires to assess for symptom change and will complete a telephone assessment to re-assess for PTSD and MDD. Participants who continue with the trial will receive the intervention and assessments at the same frequency as the initial SHAPE intervention condition. A final set of follow-up questionnaires will be sent 6 months after the participant completes the intervention as well as a final telephone assessment to re-assess for long-term outcomes of PTSD and MDD.

### Sample size {19}

The sample size for the randomised controlled trial was calculated based on 90% power, at a significance level (*α*) of 0.05, to detect a difference in proportions of the number of participants continuing to have PTSD and MDD at 8 weeks post-random allocation. An anticipated recovery rate of 50% of participants in the intervention condition (i.e. 50% not recovered) and 16% of participants in the waitlist condition (i.e. 84% not recovered) corresponds to a relative risk of 0.59, requiring 39 participants per condition (78 in total). Allowing for up to 15% attrition increases the required sample size to *N* = 92 (46 participants per condition).

The effect size (50% recovery) for the intervention condition was informed by the NHS Talking Therapies target of 50% recovery for all individuals completing treatment [36]. This is a conservative estimate as findings from the pilot study of SHAPE comparing participants scores on the PCL-5 and PHQ-9 found recovery rates of 77.1% and 64.3% for MDD and PTSD respectively (Wild J, McKinnon A, Wilkins A, Storch C, Browne H, & Ehlers A. Cognitive therapy coaching for PTSD and depression symptoms in healthcare workers repeatedly exposed to trauma: a pilot evaluation of SHAPE, under review). We chose a 50% recovery rate in line with the NHS Talking Therapies target and to allow for greater power for the secondary analyses. The effect size (16% recovery) for the waitlist condition was based on previous studies documenting natural recovery rates for PTSD and MDD over a 2–3-month period of between 12 and 20% for both disorders [37–40]. The 15% attrition estimate was based on dropout rates of similar trials and studies reporting rates of between 10 and 20% [37, 41–47].

Approximately *N* = 20 HSCWs will be recruited for the qualitative interview study or until a diversity of participants is reached. Purposive sampling will be used to recruit a wide range of HSCWs from the RCT who responded well and less well to the intervention. This will allow for a diversity of experiences and views to be collected and analysed. A sampling frame has been developed to ensure that there is a diversity of participants being interviewed based on gender, recruitment method, withdrawal status, recovery status, primary mental health condition, and time since trauma. The sample size for this study is guided by research informing recommendations for reaching information power [48] as well as being consistent with similar qualitative interview studies in this area [49, 50].

An additional *N* = 5 interviews will be conducted with wellbeing coaches who delivered the SHAPE intervention during the trial. *N* = 5 has been chosen based on the predicted number of coaches delivering the intervention during the trial who will be available to be interviewed.

### Recruitment {20}

Collaborating organisations that offer mental health support to health and social care workers across the UK including Canopi [51], a Welsh Government initiative to support the mental health of NHS workers, and The Ambulance Staff Charity (TASC) [52] will be invited to signpost potential participants to the trial. The study team will also use its existing networks to assist in reaching different groups of HSCWs around the UK. Self-referrals will be accepted where participants can find the trial information sheet and consent form through social media advertisements, from the research team’s website [53], trial registration website [54], blog posts, social media posts, and flyers distributed from snowball sampling by previous participants.

## Methods: assignment of interventions

### Randomisation

#### Sequence generation {21a and 21b}

After completing the telephone assessment, the second consent form, and the baseline questionnaires, participants will be randomised to one of two conditions (SHAPE or waitlist condition) on a 1:1 ratio. Randomisation will be conducted using the computer software programme Minim [55] by an independent researcher, separate to the main research team to minimise potential researcher bias. The software uses a minimisation method, and participants will be randomised based on 3 potential prognostic factors (absence/presence of PTSD using the SCID-5, depression symptom severity on the PHQ-9 (<15 or 15), and time since trauma (>/<18 months) on the PCL-5). We chose to use a median split of 15 for MDD severity using the PHQ-9 based on our pilot study where the median score on the PHQ-9 for people who were diagnosed with MDD using the SCID-5 was 15. This decision is consistent with a recent research study that dichotomised participants on the PHQ-9 with 0 for those scoring <15 or 1 for those scoring 15 or more [56]. For participants who have not experienced a traumatic event in their lives and/or do not meet criteria for PTSD, they will be asked to complete the PCL-5 in relation to the most stressful event that currently bothers them the most, indicating when this occurred. This event will be used for the time-since-trauma prognostic factor.

#### Allocation concealment mechanism {22}

The lead researcher will enter the three minimisation variables for an eligible participant into a secure excel sheet and notify the independent researcher linked to the study to randomise the participant. Once the independent researcher randomises the participant, the lead researcher will be notified of this and can then inform the participant of their condition allocation over email. Randomisation will take place in the order of when a participant completes their baseline measures.

#### Implementation {23}

The independent researcher linked to the trial will generate the allocation sequence and randomly assign participants to conditions. They will have access to a condition assignment table that other members of the research team will not be able to access, apart from the lead researcher (JL), until data collection has concluded and all participants have completed the intervention or waitlist condition. The lead researcher will be notified once a participant has been randomised and will inform the participant and their assigned coach of this outcome.

## Blinding

### Who will be blinded after assignment to interventions {24a}

Participants and coaches will not be blind to treatment allocation due to the nature of the intervention. Assessors conducting the SCID-5 assessment for treatment outcome will be blind at all post-randomisation assessment timepoints. Members of the trial steering committee will also be blind for the duration of the trial until all data analyses have been completed.

### How blinding will be achieved {24b}

Assessors conducting the post-treatment allocation assessments will be blind for the duration of the study until data lock. The research team will request participants to refrain from disclosing group allocation during post-randomisation assessments. Assessors will be instructed to refrain from asking about group allocation during assessments.

### Procedure for unblinding if needed {24c}

Assessors will be unblinded if a risk and safeguarding issue arises during an assessment and the study team deem that it is in the best interests for the assessor to be aware of what condition the participant was assigned to after the assessment has been completed.

## Methods: data collection, management, and analysis

### Plans for assessment and collection of trial data {25a}

Outcome data will be collected from all participants, including participants who withdraw from the intervention wherever possible. Furthermore, participants will be thanked with an honorarium of £20 once they complete their second SCID-5 assessment to improve data collection and completeness. In addition, for participants who withdraw or who are difficult to contact, multiple efforts will be made by the research team to reach these participants and invite them to complete measures at all data collection timepoints. Attempts will be made through different modalities (i.e. email, telephone, SMS) in case participants change contact information or are more accessible through one modality. The research team will continue to contact hard-to-reach participants throughout the trial until they complete outcome data or they withdraw consent from being sent outcome measure invitations. Shortened versions of questionnaires will also be made available to participants to complete, which will contain the most important measures (i.e. PCL-5 and PHQ-9) to reduce the potential burden on participants who struggle to complete follow-up measures. An additional monetary incentive (£10 e-voucher) for filling out a shortened questionnaire (PCL-5 and PHQ-9) will be issued for particularly hard-to-reach participants who do not respond to any contact method and who have not completed either the self-report measures or SCID-5 assessment at any post-randomisation timepoint. Most of the outcome data will be collected using Qualtrics [57], a widely used data collection online software programme. Participant outcome data on Qualtrics will be pseudonymised by participant ID. Access to the outcome data will be restricted to the main study team (JL, JW, CC, AE, and AM) using a secure login (two-factor authentication). To minimise data entry errors and ensure completeness, data validation procedures including force response settings and single-choice restrictions will be implemented within the Qualtrics survey platform where applicable. The only data that will not be collected over Qualtrics is the SCID-5 assessment measures collected at baseline, 8 weeks post-random allocation, and 6 months post-intervention completion as well as the semi-structured interviews. This data will be in the format of interview recordings, online SCID-5 assessment sheets, and online topic guides that will be safely stored in restricted access folders on Nexus 365 OneDrive for Business, only accessible by the main study team. Data completeness and anomalies will be checked on a weekly basis by the trial lead (JL), and any issues will be flagged and brought to weekly supervision with the PI (JW) to be resolved on a case-by-case basis.

### Measures

#### Primary outcome measure

Structured Clinical Interview for DSM-5 (PTSD and MDD modules) [22]. The SCID-5 is a semi-structured diagnostic interview for DSM-5 disorders and demonstrates good to excellent reliability, specificity, and validity [22]. The trial will use the SCID-5 PTSD and MDD modules. The SCID-5 PTSD module consists of 32 questions, whilst the SCID-5 current MDD module consists of 14 questions. The SCID-5 for PTSD and MDD will be administered by a trained assessor at 8 weeks post-random allocation.

#### Secondary outcome measures

Structured Clinical Interview for DSM-5 (PTSD and MDD modules) [22]. All participants, including those initially assigned to the 8-week waitlist condition, will be invited to be re-assessed for PTSD and MDD with the SCID-5 at 6 months after completing the SHAPE intervention.

Posttraumatic Stress Disorder Checklist for DSM-5 (PCL-5) [20]. The PCL-5 is a 20-item self-report questionnaire that provides a continuous measure of the severity of PTSD symptoms. Items align with the DSM-5 criteria for PTSD. Items are rated on a 5-point Likert scale (0 = not at all, 4 = extremely) and summed to provide a total score of PTSD symptom severity. Scores range from 0 to 80, with higher scores reflecting more severe PTSD symptoms. The PCL-5 is a psychometrically robust measure [20, 23]. Participants in the intervention condition will complete the PCL-5 at baseline, during the intervention (weeks 1–7), at 8 weeks post-random allocation, and at 6 months post-intervention completion for secondary analyses. Participants in the waitlist condition will complete the PCL-5 at baseline, 4 weeks, and 8 weeks post-random allocation. Waitlist participants who subsequently receive the SHAPE intervention will complete the PCL-5 at the same timepoints during and after the intervention as the intervention group.

Patient Health Questionnaire (PHQ-9) [21]. The PHQ-9 is a 9-item self-report questionnaire that provides a continuous measure of MDD symptom severity. Items are rated on a 4-point Likert scale (0 = not at all, 3 = nearly every day), and responses are summed together to reflect an overall score of MDD symptom severity. Scores range from 0 to 27, with higher scores reflecting more severe depression symptoms. The PHQ-9 demonstrates good reliability and validity [21]. Participants will be assessed for MDD symptom severity with the PHQ-9 at the same time points as for the PCL-5 for each condition respectively.

Generalised Anxiety Disorder Scale (GAD-7) [28]. The GAD-7 is a seven-item self-report questionnaire that provides a continuous measure of anxiety symptom severity. Items are rated on a 4-point Likert scale (0 = not at all, 3 = nearly every day), and responses are summed for an overall score. Scores range from 0 to 21, with higher scores reflecting more severe symptoms. The GAD-7 is psychometrically sound [24]. Participants will be assessed for GAD using the GAD-7 at baseline, 4 weeks post-random allocation, 8 weeks post-random allocation, and at 6 months post-intervention completion for secondary analyses.

Insomnia Sleep Index (ISI) [25]. The ISI is a seven-question self-report measure that provides a continuous measure of sleep quality. Items are rated on a 5-point Likert scale (0 = none, 4 = very severe), and responses are summed together to reflect an overall score of sleep quality. Scores range from 0 to 28, with higher scores reflecting poorer sleep. The ISI demonstrates excellent reliability and validity [25]. Participants will be assessed for insomnia symptoms at baseline and at 8 weeks post-random allocation for secondary analyses.

International Trauma Questionnaire (ITQ) [27]. The ITQ is an 18-item self-report questionnaire that provides a categorical and dimensional scoring system to assess PTSD and C-PTSD diagnosis and symptom severity respectively. Response items are on a 5-point Likert scale (0 = not at all, 4 = extremely). The measure has two major subscales consisting of three symptom clusters, the Posttraumatic Stress Disorder Subscale (items 1–6) and the Disturbances in Self-Organisation Subscale (items 10–15). Dimensional scores of 0–24 can be calculated for the two major subscales by summing the items to assess severity. The ITQ has been validated by Hyland et al. [26]. Cloitre et al. [27] established the optimal symptom indicators of C-PTSD and PTSD by item response theory analysis. Participants will be assessed for C-PTSD symptom severity with the ITQ at baseline, 8 weeks post-random allocation, and 6 months post-intervention completion for secondary analyses.

#### Process measures

Responses to Intrusions Questionnaire Short Version (RIQ-s) [29]. The RIQ-s is a 12-item self-report questionnaire, which assesses the frequency of maladaptive responses to unwanted memories. A total score can be calculated as well as scores for each of the three subscales: thought suppression (items 1–3), rumination (items 4–9), and numbing (items 10–12) [28, 29]. Items are rated on a 4-point scale (0 = never, 3 = always). The RIQ-s demonstrates good reliability and predictive validity [30]. Participants will complete the RIQ-s at baseline, 4 weeks post-random allocation, 8 weeks post-random allocation, and at 6 months post-intervention completion for secondary analyses.

Posttraumatic Cognitions Inventory Short Version (PTCI-s) [31]. The PTCI-s is a 20-item self-report questionnaire that assesses negative trauma-related appraisals representing cognitive themes. The cognitive themes include vulnerable self, self-criticism, overgeneralised danger, preoccupation with unfairness, perceived permanent change, alienation, hopelessness, shame, and a negative view of one’s body. Items are rated on a 7-point scale (1 = totally disagree, 7 = totally agree). A total score is the sum of all the items, with a lower score indicating better clinical outcomes [31]. The PTCI-s demonstrates good reliability and predictive validity [31]. Participants will complete the PTCI-s at baseline, 4 weeks post-random allocation, 8 weeks post-random allocation, and at 6 months post-intervention completion for secondary analyses.

#### Wellbeing and resilience measures

Warwick-Edinburgh Mental Wellbeing Scale (WEMWBS) [32]. The WEMWBS is a 14-item self-report questionnaire of wellbeing. Items are rated on a 5-point scale (1 = none of the time, 5 = all of the time). A total score is the sum of all the items, with a higher score indicating greater mental wellbeing [32]. The highest score a participant can obtain on this scale is 70, whilst the lowest is 14. The WEMWBS has good psychometric properties [32, 33]. Participants complete this measure at baseline, 8 weeks post-random allocation, and at 6 months post-intervention completion.

Connor-Davidson Resilience Scale 25 (CD-RISC-25) [34]. The CD-RISC-25 is a 25-item self-report questionnaire measuring psychological resilience. Items are rated on a 5-point scale (0 = not true at all, 4 = true nearly all the time). Scores range from 0 to 100, with higher scores reflecting greater resilience [34]. The CD-RISC-25 demonstrates good reliability and predictive validity [34]. The CD-RISC-25 will be given at baseline, 8 weeks post-random allocation, and at 6 months post-intervention completion.

Social support scales (adapted from Sarason et al. [35]). Two social support scales will measure perceived support from (i) family and friends and (ii) colleagues and superiors at work. The scale for friends and family is a 7-item self-report questionnaire with items rated on a 7-point scale (1 = never, 7 = always). The scale for colleagues and superiors is a 6-item self-report questionnaire with items rated on a 7-point scale (1 = never, 7 = always). A higher score on both scales indicates more positive perceptions of social support. Perceived social support will be assessed at baseline, 8 weeks post-random allocation, and at 6 months post-intervention completion.

#### Life and job satisfaction questions

Two questions ask participants to rate how much on a scale of 0–100 (0 = not at all; 100 = extremely) they are satisfied with their life and job at baseline, mid-intervention (week 4), 8 weeks post-random allocation, and 6 months intervention follow-up.

#### Previous trauma measures

Adverse Childhood Experiences Revised Questionnaire (ACE-Q) [58]. The ACE-Q is a 10-item self-report questionnaire that is used to quantify the number of adverse experiences a person has experienced before their 18th birthday. Items are dichotomous, rated as being yes or no. The total score is the number of yes responses. A higher score indicates that a person has experienced a greater number of childhood traumatic events. The ACE-Q has good psychometric properties [58]. The ACE-Q will be administered at baseline.

Life Events Checklist for DSM-5 (LEC-5) [59]. The LEC-5 is a 17-item self-report measure used to screen for the types of traumatic events that may have occurred in a person’s life. The response options are as follows: happened to me; witnessed it; learned about it; part of my job; not sure; doesn’t apply. Respondents can select multiple choices for each question. The LEC-5 is scored as the number of different types of trauma a person has experienced. The LEC-5 demonstrates adequate psychometric properties [60]. Participants will complete the LEC-5 at baseline.

New trauma experienced questions. Two questions will assess whether participants have experienced new stressful or traumatic events, including the nature of any such events at 4 weeks post-random allocation, 8 weeks post-random allocation, and at 6 months post-intervention completion. The purpose of these questions is to account for new trauma and its potential influence on outcome.

#### Questionnaire instruction alterations for timeframe

For the weekly administration of measures during the intervention period (weeks 1–7), we modified the PCL-5 to refer to symptoms experienced *in the past week* rather than *in the past month*. We made a similar modification to the PHQ-9 and GAD-7 for weeks 1–7 and weeks 4 and 8 respectively to refer to the experience of symptoms *over the last week* rather than *over the last two weeks*. For all other secondary measures (ISI, ITQ, RIQ-s, PTCI-s, WEMWBS, CD-RISC-25, and social support scales), we have changed the timepoint to be *in the past week* compared to the usual timepoints for these measures for weeks 4 and 8. The baseline and 6 months post-intervention follow-up assessment timepoints will use the standard timepoints for all measures.

#### Health economic measures

Health economics outcome measures are included in the trial to assess for resource utilisation (Client Service Receipt Inventory (CSRI) [61]), productivity loss (iMTA Productivity Cost Questionnaire (iPCQ) [62]), quality of life (EuroQol 5 Dimensions 5 Levels health survey (EQ-5D-5L) [63] and Recovering Quality of Life Questionnaire (Re-QoL) [64]), and health problems (Short Form Health and Labour Questionnaire (SF-HLQ) [65]). These measures were chosen with the assistance of a health economist, and some of the measures have been shortened due to overlapping questions. Health economic measures will be administered at baseline, 8 weeks post-random allocation, and at 6 months post-intervention follow-up for secondary analyses. Between-group differences will be measured at 8 weeks post-random allocation for quality of life, and within-group analyses including everyone who received the SHAPE intervention will be conducted at 6 months post-intervention completion to compare productivity loss, resource utilisation, and health problems from pre- to post-intervention. Results will be published separately from the primary and secondary psychological outcomes.

#### Qualitative feedback

Semi-structured interviews exploring participants’ and coaches’ experiences of the SHAPE intervention. This complementary study aims to collect constructive feedback from participants and coaches about the perceived acceptability, accessibility, and suitability of SHAPE to guide improvements. Approximately 20 participants and 5 coaches will be individually interviewed remotely (by a phone call or by audio link on Microsoft Teams), with interviews lasting up to 1 h. The interviews will be audio recorded with participants’ consent and transcribed verbatim. Data of interest will focus on what worked and what worked less well from the provider’s and receiver’s point of view. Interview questions will be designed and developed with the help of PPIE and will include questions about the training, coaching, tools used, online modules, accessibility, feasibility, and suitability of SHAPE. Interviews conducted with trial participants will occur during the period between participants completing the SHAPE intervention and receiving their 6-month follow-up. Interviews with wellbeing coaches will take place during the period that coaches are delivering the SHAPE intervention, or shortly afterward.

### Plans to promote participant retention and complete follow-up {25b}

To support retention and prevent dropouts, participants and prospective participants will receive a £10 e-voucher as an honorarium for each SCID-5 assessment they complete (4 assessments for the waitlist condition, 3 assessments for the intervention condition, and 1 assessment for prospective participants found to be ineligible). Participants will be able to spend their e-voucher on a variety of brands.

Participants are allowed to withdraw at any stage of the trial and ask for their data to be deleted. Their future treatment in NHS and private services will not be affected by withdrawing from the study. Withdrawal after randomisation will be recorded along with reasons (if provided). Participants who withdraw from the intervention or waitlist condition will continue to be sent follow-up questionnaires unless they withdraw their consent for this.

Participants who do not respond to initial invitations to take part in SCID-5 assessments will be contacted using a variety of available approaches (email, phone call, SMS) unless they have withdrawn their consent from the trial. Participants who are reluctant to complete the longer questionnaires or the SCID-5 assessments at 4 weeks post-random allocation, 8 weeks post-random allocation, or 6 months post-intervention completion will be sent the PCL-5 and PHQ-9 as a minimum dataset.

### Data management {26}

The trial will comply with the Data Protection Act that requires research data to be pseudonymised as soon as it is practical to do so. The research team will ensure that participant confidentiality is maintained and protected. Participants will each be assigned a four-digit unique identifier to protect their privacy. This unique ID will be attached to all participant outcome data instead of using the participant’s personal information. A linkage list connecting personal data (i.e. name, email address, mobile number, Trust, occupation, and referral pathway) to unique ID will be kept separate from the trial data and will be safely stored in a separate folder on a 2-factor authenticated restricted access file on the University’s secure storage space, Nexus 365 OneDrive for Business, that only the trial lead will be able to access.

Once a participant has completed the trial, and all data have been analysed, personal data (first and last name, age, index event, employment organisation, and health economics information) including contact details (unless the participant gives permission for us to retain these) and any identifiable information about physical and mental health will be destroyed.

Participants’ electronic consent forms will be uploaded securely and stored in a separate file on Nexus 365 OneDrive for Business. Records of consent will be retained for a minimum of 3 years after the end of the research study. If participants complete a telephone assessment, the audio recording will be uploaded onto Nexus 365 OneDrive for Business, and the original recording will be deleted. These recordings will be stored securely until participants have completed the study and their data have been analysed. The recordings will then be permanently deleted.

Raw outcome data (questionnaires, consent forms, screening measures) uploaded to Outlook 365 for Business will be protected. Outlook 365 for Business uses a 2-factor authentication method and is secure. Additionally, the University of Oxford implements up-to-date procedures to keep its accounts well protected and safe.

Raw data stored on Qualtrics will be protected through Qualtrics high-end security measures as well as using a secure password that will be generated by a high-end password manager. Qualtrics is a highly secure software platform that prioritises the protection and safety of customer data. Qualtrics servers are protected by high-end firewall systems and Qualtrics uses Transport Layer Security (TLS) encryption for all transmitted data. Once the final data have been analysed and the study is complete, all questionnaires and participant data on Qualtrics will be destroyed.

An interim analysis will be conducted during the trial to allow for the primary and secondary analyses (excluding the 6-month analyses) to be conducted, written up, and presented in a DPhil thesis. This thesis will be embargoed for 3 years. Due to the trial being deemed to be low risk, this interim analysis will not dictate how the rest of the follow-up is performed and will not result in the trial being terminated. For the interim and final statistical analyses, the pseudonymised data will be downloaded from Qualtrics and stored on a password-protected file on Nexus 365 OneDrive for Business. Data files downloaded from Qualtrics will be cross checked to ensure that the number of rows (participants) and columns (questionnaire items) matches the original number of submissions and questionnaire items respectively, on Qualtrics. Changes made to the trial data will be logged in an audit log. The final data files of the anonymised data will be available to the research team, principal investigator, and health economist.

De-identified research data will be stored on Nexus 365 OneDrive for Business for the duration of the trial. De-identified data will be stored here for 3 years after the end of the research project and then will be permanently deleted.

## Statistical methods

### Statistical methods to compare groups for primary and secondary outcomes, including harms {27a and 27b}

Consistent with the BMJ and Consolidated Standards of Reporting Trials guidelines, all analyses will be intent-to-treat (ITT) from randomisation, whereby participants will be analysed under the same conditions as they were randomly assigned, irrespective of the treatment they actually received, subsequent willingness, adherence, or completion of follow-up outcome measures. Primary and secondary main trial analyses will be conducted once all willing participants in both conditions have completed their 8-week post-random allocation SCID-5 assessment and questionnaires. Follow-up analyses will be conducted at the conclusion of the trial period (i.e. after all willing participants in both conditions have completed their 6-month post-intervention follow-up SCID-5 assessment and questionnaires).

#### Main trial analyses

##### Primary outcome

The primary outcome measure will be SCID-5 diagnosed PTSD and MDD at 8 weeks post-random allocation. The primary outcome measure will be analysed using logistic regression. The outcome will be a binary outcome measure whereby, if a participant meets diagnosis for one or both disorders (PTSD and/or MDD), they will score a 1 (present), whereas if a participant does not meet diagnostic criteria for either disorder, they will score a 0 (absent). All participants will begin the trial with a score of 1 since they will have a diagnosis of PTSD and/or MDD at the outset, consistent with eligibility criteria.

Where the primary outcome is measured at one time point, it has been shown that logistic regression is valid with a data missing at random assumption [66]. Complete case analysis will be conducted if missingness is <5%, whereby participants with missing data will be excluded from the primary analysis. Multiple imputation will be conducted as a sensitivity analysis for the primary outcome if missingness is >5% to check for robustness of results.

Models will include fixed factors of condition (SHAPE, waitlist). The stratification variables absence/presence of PTSD assessed with the SCID-5 at baseline, depression symptom severity (<15 or 15) assessed at baseline with the PHQ-9, and time since reported trauma (>/<18 months) assessed at baseline with the PCL-5 will be included as fixed covariates. The significance level will be set to *p* < 0.05 for comparison between the SHAPE and waitlist conditions.

##### Secondary outcomes

All secondary outcome analyses will be treated as exploratory analyses.

Between-group analyses of the continuous secondary outcome measures will include mixed effects regression models and multiple regression models following the same specifications as the primary outcome measure with additional fixed effects for time, a time by condition interaction, and a random effect for participant for the mixed-effect regression models. Mixed effects regression models allow for the inclusion of all available data from randomised participants (ITT), can account for missing data, and can account for repeated measures [67]. Between-group effect sizes (Cohen’s *d*) will be calculated by dividing the adjusted group difference by the baseline standard deviation.

For secondary outcome measures collected at 4 and 8 weeks post-random allocation (i.e. PCL-5, PHQ-9, GAD-7, PTCI-s, and RIQ-s), statistical models of treatment effects will include categorical fixed factors of time (4 weeks, 8 weeks post-random allocation), condition (SHAPE, waitlist), and the time × condition interaction. The interaction permits the estimation of differences between treatments at each timepoint. Participant will be treated as a random effect to account for between-participant variation.

For secondary outcome measures collected at 8 weeks post-random allocation only (i.e. ISI, ITQ, WEMWBS, CD-RISC, and social support scales), we will fit multiple regression models including condition (SHAPE, waitlist).

The stratification variables absence/presence of PTSD assessed with the SCID-5 at baseline, depression symptom severity (<15 or 15) assessed at baseline with the PHQ-9, and time since reported trauma (>/<18 months) assessed at baseline with the PCL-5 will be included as fixed covariates, along with the baseline score of the measure being analysed. Significance level will be set to *p* < 0.05 for comparison between the SHAPE and waitlist conditions.

To assess for potential correlates of PTSD and MDD symptom change, we will conduct correlational analyses to assess the strength of the relationship between change in posttraumatic cognitions and maladaptive responses to intrusions from baseline to week 4, and from baseline to week 8 and change in PTSD and MDD symptom severity over the same time periods, controlling for baseline scores on these measures. This analysis will be conducted with the whole sample at week 8 to include post-intervention and post-wait participants.

#### Follow-up analyses

To measure the long-term effects of the SHAPE intervention, secondary analyses will be conducted for all participants who received the intervention (i.e. including post-waitlist participants going on to receive SHAPE). Secondary outcome measures for these analyses will include dichotomous SCID-5 diagnosed PTSD and MDD at 6 months post-intervention completion, and continuous symptom, resilience, and wellbeing measures collected at 6 months post-intervention completion.

To test if there is a significant difference between receiving SHAPE immediately or after waitlist, between-group analyses of the dichotomous and continuous secondary outcome measures at 6 months post-intervention completion will first be conducted. If there are no significant group differences, within-group analyses will be conducted including everyone who received the intervention. Alternatively, if there are significant group differences, separate within-group analyses will be conducted for each condition. Since participants randomised to the two conditions receive the SHAPE intervention at different starting points (i.e. immediately versus post-wait), time will therefore be coded in relation to the start of the intervention rather than time since randomisation for these analyses. Timepoints will therefore include pre-intervention (baseline or post-wait scores), post-intervention (after 8 weeks of intervention), and 6 months post-intervention completion.

For the dichotomous secondary outcome measure, SCID-5 diagnosed PTSD and MDD at 6 months post-intervention completion, a mixed effects logistic regression model will be used. For the between-group analysis, the model will include fixed factors of time (post-intervention, 6-month post-intervention completion), condition (SHAPE, waitlist), and time × condition. Participant will be treated as a random effect. If there is not a significant difference between groups (receiving SHAPE immediately or after waitlist), a within-group analysis will be conducted that will include fixed factors of time (post-intervention, 6-month post-intervention completion), and participant will be treated as a random effect. If there is a significant difference between groups, separate within-group analyses will be conducted for each condition. The stratification variables absence/presence of PTSD based on the SCID-5, depression symptom severity (<15 or 15) based on the PHQ-9, and time since trauma (>/<18 months) based on the PCL-5 will be included as fixed covariates in all models, along with the pre-intervention diagnosis of PTSD and MDD.

For the continuous secondary outcome measures collected at 6 months post-intervention completion, mixed effects regression models will be used. For the between-group analyses (receiving SHAPE immediately or after waitlist), models will include fixed factors of time (post-intervention, 6-month post-intervention completion), condition (SHAPE, waitlist), and time × condition. Participant will be treated as a random effect. If there is not a significant difference between groups, within-group analyses will be conducted that will include fixed factors of time (post-intervention, 6-month post-intervention completion), and participant will be treated as a random effect. If there are significant differences between groups, separate within-group analyses will be conducted for each condition. The stratification variables absence/presence of PTSD based on the SCID-5, depression symptom severity (<15 or 15) based on the PHQ-9, and time since trauma (>/<18 months) based on the PCL-5 will be included as fixed covariates in all models, along with the pre-intervention score of the measure being analysed.

A detailed statistical analysis plan is available to view as a supplementary file.

### Methods in analysis to handle protocol non-adherence and any statistical methods to handle missing data {27c}

For the primary outcome, if missing data is <5%, this will be handled with complete case analysis whereby participants with missing data at the primary outcome timepoint (8 weeks post-randomisation) will be excluded from the analysis. If missing data is >5%, multiple imputation will be employed.

Due to the secondary analyses being exploratory in nature, no sensitivity analyses will be conducted to test assumptions for missing data. However, the statistical analysis methods used for the secondary measures are able to handle missing data under a data missing at random assumption.

More information on handling protocol non-adherence and missing data is included in the statistical analysis plan [68].

### Methods for additional analyses (e.g. subgroup analyses) {27d}

#### Health economic evaluation

A health economics evaluation will compare the costs involved in administering SHAPE and the costs saved in terms of patient quality of life, working hours, and use of healthcare services. Health economic measures including the iMTA Productivity Cost Questionnaire short (iPCQ) [62], Short Form Health and Labour Questionnaire (SF-HLQ) [65], Client Service Receipt Inventory (CSRI) (*edited*) [61], Quality of Life (EQ-5D-5L) [63], and Recovering Quality of Life (Re-QoL) [64] will be collected at baseline (0 weeks), 8 weeks post-random allocation, and at 6 months post-intervention follow-up. Results will be expressed in costs per group.

#### Qualitative evaluation

Transcripts will be transcribed verbatim, uploaded onto NVivo version 15, and indexed using the framework analysis method [69, 70]. Framework analysis will be used to identify key themes, and findings will be written up alongside the quantitative findings from the RCT [71]. The framework analysis method was chosen since it is a dynamic, grounded, and systematic approach of organising data in a structured way. It allows for between and within case analysis, enabling comparison between, and associations within, cases to be made [72]. We hope this analysis method will allow for our findings to be easily understood by individuals from different disciplinary backgrounds due to its structured and accessible design.

## Methods: monitoring

### Composition of the data monitoring committee, its role and reporting structure {28a}

Not applicable. The TSC will assume the role of the DMC as the trial is deemed to be low risk. There are no stopping rules in place for this trial since it does not use Investigational Medicinal Products (non-CTIMP). Furthermore, there are no foreseen associated risks with completing the questionnaires, assessments, or the intervention.

### Interim analyses {28b}

An interim analysis will be conducted once all available 8-week data have been collected from participants. The results of this interim analysis will not change how the rest of the follow-up is performed, and the trial will not be terminated as it is considered low risk. The results will not be shared with participants, coaches, or assessors until all follow-up data have been collected.

### Frequency and procedures for monitoring trial conduct {29}

The principal investigator will monitor trial conduct and data collection on a weekly basis. Researchers involved in various aspects of the trial will report their progress in various ways. For coaches delivering the intervention, they will complete a coach log after each call outlining what they covered in the call and the time taken for the call and administration. For assessors conducting clinical assessments, they will be required to upload their assessment sheets and a recording of the assessment into a secure shared folder on Nexus 365 OneDrive for Business that only the research team will be able to access. Coaches and assessors will be able to bring any issues or concerns to weekly supervision with a member of the research team.

## Ethics

### Research ethics approval {30}

This study was approved on December 7, 2022, by the Medical Sciences Interdivisional Research Ethics Committee at the University of Oxford, ref R80469/RE008. All participants will give online consent prior to taking part in the trial at two stages, consent to initial assessment and consent to participate in the trial, which will be obtained online by the trial lead. The trial will be conducted in compliance with the principles of the Declaration of Helsinki (1996) and the principles of Good Clinical Practice.

### Plans for communicating important protocol amendments to relevant parties (e.g. trial participants, ethical committees) {31}

Any proposed changes to the protocol will be first discussed within the main research team and TSC. If approved, they will be taken to the ethics committee for final approval. Once approved, these changes will be documented in the pre-registration form online, and study material will be updated. Participants who are signing up for the trial at the time will be informed of these new changes.

### Who will obtain informed consent? {32a}

This randomised controlled trial follows the most up-to-date version of the Declaration of Helsinki. Informed consent will be taken at two time points in the study. The first timepoint is after prospective participants have read the participant information sheet, had sufficient time to consider the study, and ask any questions they may have. The first consent form will be made available remotely to complete online. The second timepoint for taking consent will be after a prospective participant has completed the screening measures and received a structured clinical assessment with a trained assessor. If a participant is eligible to take part in the trial, the lead researcher of the study will send a link to a second online consent form that carefully outlines the participant’s rights and what the study will involve. Both consent forms must be obtained before any intervention is administered to prospective participants.

### Additional consent provisions for collection and use of participant data and biological specimens in ancillary studies, if applicable {32b}

A complementary qualitative interview study will run alongside the trial to assess the suitability, acceptability, and accessibility of the intervention. If a participant or coach is invited to take part in this study, they will be sent an information sheet. If a participant or coach agrees to take part in the study, the lead researcher will invite them to complete a remote consent form before participating in the interview. A copy of the completed consent form will be emailed after they have finished the interview.

We will not be collecting biological specimens in this study.

### Confidentiality {33}

Personal information collected from prospective and enrolled participants will be stored on a secure, two-factor authenticated file only accessible by the main research team. Raw personal data will be kept in this folder for the duration of the trial until all results have been analysed and published, and then personal data will be destroyed. Personal data will be retained beyond this period only if participants provide consent to be contacted for future studies. Participants will be assigned a unique identification number for pseudonymity, and all future data collected will be assigned under their unique ID in separate folders. Participants will not be directly identifiable in any research outputs from this trial unless consent is provided (i.e. consent to provide a testimonial of their experience of the SHAPE intervention).

### Ancillary and post-trial care {34}

No provisions for post-trial care will be provided as the research trial and intervention are deemed to be low risk. Instead, participants will be able to return to treatment-as-usual if they require treatment after completing the trial.

## Discussion

This trial will be the first to evaluate the efficacy of a novel cognitive therapy coaching intervention (*SHAPE*) to treat PTSD and MDD tailored for HSCWs in the UK. The study aims to investigate the efficacy of the SHAPE intervention by comparing outcomes in the intervention condition to a waitlist condition. The study also aims to determine whether the SHAPE intervention is suitable, acceptable, and accessible to health and social care workers receiving the intervention and the coaches delivering it.

Several potential clinical implications could arise from this study should the trial find superiority of the SHAPE intervention compared to the waitlist condition. SHAPE coaching typically takes half the amount of time to complete compared to face-to-face trauma-focused therapy, which may be more accessible for HSCWs with busy or irregular schedules. SHAPE may therefore offer a cost-effective, scalable, low-intensity intervention for HSCWs with PTSD and/or MDD. Evidence from the pilot study conducted during the COVID-19 pandemic demonstrated that coaches who were working as psychological wellbeing practitioners (PWP) or psychology assistants achieved similar outcomes as clinical psychologists who administered SHAPE (Wild J, McKinnon A, Wilkins A, Storch C, Browne H, & Ehlers A. Cognitive therapy coaching for PTSD and depression symptoms in healthcare workers repeatedly exposed to trauma: a pilot evaluation of SHAPE, under review). Finally, SHAPE may constitute a highly acceptable intervention for healthcare workers since it offers personalised tools targeting modifiable risk and maintaining factors specific to this workforce as well as self-paced online learning modules to consolidate learning.

Despite the potential advantages of the SHAPE intervention, a limitation of the trial is that it compares SHAPE to an 8-week wait period rather than an alternative intervention, a design which may overestimate treatment effects since there is no control for non-specific factors such as a positive therapeutic relationship. Some research suggests that participants assigned to wait conditions avoid attempting behaviour change on their own and, as such, demonstrate limited improvement [73]. Additionally, choosing to have the primary timepoint as 8 weeks post-random allocation might be viewed as a short, controlled end point, as some participants might take longer to implement the tools and experience benefits. Finally, not being able to compare condition-related differences at 6-month follow-up prevents us from determining whether changes at this time point are intervention-specific. However, since a proportion of individuals with PTSD will experience improvement in their symptoms over time, the waitlist comparison allows us to determine intervention-specific effects and collect data on rates of natural recovery of PTSD and MDD over an 8-week period.

## Trial status

Recruitment for this trial began on February 24, 2023, and is expected to run until mid-November 2024. This protocol was submitted to the journal on 1 November 2024. We aim to complete data collection for this trial by December 2025. Protocol version 1.3, October 20, 2025.

## Supplementary Information

Supplementary Material 1.

Supplementary Material 2.

Supplementary Material 3.

## Data Availability

Trial materials can be obtained from the first author (JL). Participants in this study have given permission for their aggregated anonymised data to be shared with a data repository, such as Ox-data or the UK data archive. For participants who consent to their raw research data to be given to other researchers, including those working outside of the UK and the EU, to be used in other research studies, this will be available upon request from Prof Jennifer Wild (jennifer.wild@psy.ox.ac.uk).

## Abbreviations

CT: Cognitive therapy
CBT: Cognitive behavioural therapy
CT-PTSD: Cognitive therapy for PTSD
PCF-1: Participant consent form 1
PCF-2: Patient consent form 2
PCL-5: PTSD Checklist for DSM-5
PHQ-9: Patient Health Questionnaire
SCID-5: Structured Clinical Interview for DSM-5 (PTSD and MDD modules)
GAD-7: Generalised Anxiety Disorder Scale
ISI: Insomnia Sleep Index
PTCI-s: Posttraumatic Cognitions Inventory short version
RIQ-s: Responses to Intrusions Questionnaire short version
LEC-5: Life Events Checklist for DSM-5
ITQ: International Trauma Questionnaire
ACE-Q: Adverse Childhood Experiences Revised Questionnaire
WEMWBS: Warwick-Edinburgh Mental Wellbeing Scale
CD-RISC-25: Connor-Davidson Resilience Scale 25
iPCQ iMTA: Productivity Cost Questionnaire
SF-HLQ: Short Form Health and Labour Questionnaire
CSRI: Client Service Receipt Inventory
Re-QoL: Recovering Quality of Life Questionnaire
TSC: Trial Steering Committee
