# Supplementary material for: Supporting Healthcare and Paramedic Employees (SHAPE)—study protocol for a single-blind, superiority randomised controlled trial evaluating cognitive therapy coaching for PTSD and MDD for health and social care workers in the United Kingdom
Source: Trials. 2025 Dec 20;27:83. doi: 10.1186/s13063-025-09341-4 (PMC12838460; doi:10.1186/s13063-025-09341-4)
Supplement: Supplementary file 1 — Supplementary Material 1. [file 13063_2025_9341_MOESM1_ESM.docx]

**PARTICIPANT CONSENT FORM: Stage 1**

**Consent for Assessment**

Central University Research Ethics Committee (CUREC) Approval Reference: R80469/RE008

**Supporting Hospital and Paramedic Employees (SHAPE) with Cognitive & Behavioural Coaching for PTSD and Depression: A Randomised Controlled Trial**

**Purpose of Study:** To evaluate a brief intervention to reduce PTSD and depression in health and social care staff.

|  |  | *Please check each box* |
| --- | --- | --- |
| 1 | I confirm that I have read and understand the information sheet version ____ dated ________________ for the above study. I have had the opportunity to consider the information, ask questions and have had these answered satisfactorily. | \|  \| \| --- \| |
| 2 | I understand that my participation is voluntary and that I am free to withdraw at any time, without giving any reason, and without penalty. | \|  \| \| --- \| |
| 3 | I understand who will have access to my personal data provided, how the data will be stored and what will happen to the data at the end of the project. | \|  \| \| --- \| |
| 4 | I understand how to raise a concern or make a complaint. | \|  \| \| --- \| |
| 5 | I consent to my telephone interviews with the research psychologist being audio recorded. | \|  \| \| --- \| |
| 6 | I understand that all information will be kept strictly confidential except in rare circumstances in which it is judged that I am, or someone else is, at immediate risk of serious harm, or where information is requested by a court of law. |  |
| 7 | I agree to an assessment for PTSD or depression if my questionnaires suggest this is indicated. |  |
| **Optional:** | I agree that my personal contact details can be retained in a secure database so that the researchers can contact me about future studies. |  |

**Please note that you may only participate in this study if you are 18 years of age or older.**

☐ I certify that I am 18 years of age or older

**If you have read the information above and agree to participate with the understanding that the data (including any personal data) you submit will be processed accordingly, please check the relevant box below to get started.**

☐ Yes, I agree to complete screening questions with a view to taking part in the study. I understand that completion of screening does not oblige me to take part in the study, should I be eligible.
