# Supplementary material for: Supporting Healthcare and Paramedic Employees (SHAPE)—study protocol for a single-blind, superiority randomised controlled trial evaluating cognitive therapy coaching for PTSD and MDD for health and social care workers in the United Kingdom
Source: Trials. 2025 Dec 20;27:83. doi: 10.1186/s13063-025-09341-4 (PMC12838460; doi:10.1186/s13063-025-09341-4)
Supplement: Supplementary file 2 — Supplementary Material 2. [file 13063_2025_9341_MOESM2_ESM.docx]

**PARTICIPANT CONSENT FORM: Stage 2**

**Consent for Treatment**

Central University Research Ethics Committee (CUREC) Approval Reference: R80469/RE008

**Supporting Hospital and Paramedic Employees (SHAPE) with Cognitive & Behavioural Coaching for PTSD and Depression: A Randomised Controlled Trial**

**Purpose of Study:** To evaluate a brief intervention to reduce PTSD and depression in health and social care staff.

|  |  | *Please check each box* |
| --- | --- | --- |
| 1 | I confirm that I have read and understand the information sheet version ____ dated ________________ for the above study. I have had the opportunity to consider the information, ask questions and have had these answered satisfactorily. | \|  \| \| --- \| |
| 2 | I understand that my participation is voluntary and that I am free to withdraw at any time, without giving any reason, and without penalty. | \|  \| \| --- \| |
| 3 | I understand who will have access to my personal data provided, how the data will be stored and what will happen to the data at the end of the project. | \|  \| \| --- \| |
| 4 | I give permission for the aggregated anonymised data to be shared with a data repository, such as Ox-data or the UK data archive, and other responsible researchers. | \|  \| \| --- \| |
| 5 | I understand how this research will be written up and published. | \|  \| \| --- \| |
| 6 | I understand how to raise a concern or make a complaint. | \|  \| \| --- \| |
| 7 | I consent to my telephone interviews with the research psychologist being audio recorded. | \|  \| \| --- \| |
| 8 | Use of quotations: Please indicate your preference (select *one* option):   1. I do not wish to be quoted. **or** 2. I agree to the use of quotations in research outputs if I am not identifiable. **or** 3. I agree to the use of direct quotations, attributed to my name, in research outputs. | \|  \| \| --- \|  \|  \| \| --- \|  \|  \| \| --- \| |
| 9 | I understand that all information will be kept strictly confidential except in rare circumstances in which it is judged that I am, or someone else is, at immediate risk of serious harm, or where information is requested by a court of law. |  |
| 10 | I agree to take part in the study |  |
| **Optional:** | I agree for research data collected in this study to be given to researchers, including those working outside of the UK and the EU, to be used in other research studies. I understand that any data that leave the research group will be anonymised so that I cannot be identified. |  |
| **Optional:** | I agree that my personal contact details can be retained in a secure database so that the researchers can contact me about future studies. |  |
