# Supplementary material for: Supporting Healthcare and Paramedic Employees (SHAPE)—study protocol for a single-blind, superiority randomised controlled trial evaluating cognitive therapy coaching for PTSD and MDD for health and social care workers in the United Kingdom
Source: Trials. 2025 Dec 20;27:83. doi: 10.1186/s13063-025-09341-4 (PMC12838460; doi:10.1186/s13063-025-09341-4)
Supplement: Supplementary file 3 [file 13063_2025_9341_MOESM3_ESM.docx]

APPENDIX A

SHAPE – Supporting Healthcare and Paramedic Employees with Cognitive Therapy Coaching for PTSD and Depression

Safeguarding Protocol for defining and recording Adverse Events (AEs) and Serious Adverse Events (SAEs) for SHAPE Trial.

Trial Steering Committee. 

The independent Trial Steering Committee (TSC) is responsible for monitoring the progress of the study and the rights, safety, and well-being of participants, ensuring essential improvements are in place and agreeing to protocol amendments. As a part of this role, the TSC will monitor and review the acceptability of outcomes and any risks or serious adverse events that occur in any aspect of the SHAPE project (including the qualitative study).

Monitoring of SAEs and AEs is done by all involved in the studies if an event is reported by a participant.

Defining adverse events in the SHAPE project. 

For the purposes of the current studies, the definition of adverse events (AEs) has been adapted from the National Research Ethics Service (NRES) definition and will include events that: (1) result in death; (2) are life threatening; (3) require hospitalisation; (4) result in persistent or significant disability or incapacity; (5) are otherwise considered medically significant by the investigator; (6) are considered by the investigator to be a significant impairment to immediate or longer term wellbeing for an individual, family or wider community.

This study will exclude the following outcomes: 

- Non-psychiatric hospitalisation; 

- Non-psychiatric disability or incapacity; 

- AEs or serious adverse events (SAEs) that are not directly related to participation in the SHAPE studies (RCT or complementary qualitative study)

The following sections detail potential SAEs and AEs. These will be monitored and updated (in consultation with the Trial Steering Committee) throughout this study to capture any further potential SAEs or AEs that become apparent. 

SHAPE Protocol for defining and recording AEs and SAEs V1 24.09.2023

Potential Serious Adverse Events (SAEs) – reportable to TSC. 

The following events will be reported to the TSC: 

- Admission to psychiatric hospital; 

- Sectioned under the Mental Health Act; 

- Significant and sustained deterioration of pre-existing mental health condition or symptoms that require immediate intervention that cannot be accommodated within the study protocol; 

- Active suicidal behaviour with a plan; 

- Diagnosis of a new mental health condition;

- A serious safeguarding issue is revealed (i.e., harm to others). 

Potential adverse events (AEs) – not immediately reportable to TSC. 

The following AEs will be recorded, logged, and monitored by the Principal Investigator (PI) and Study Team, but not reported to the TSC (unless AEs are recurring): 

- One or more aspects of the study procedure induces unacceptable levels of distress for either the participant, or a member of the research team 

- A sustained and significant increase in detrimental behaviours (e.g., behavioural difficulties, missing work, disengagement from family or home life). 

- The emergence of new detrimental behaviours (e.g. self-harm, safety seeking behaviours, behavioural difficulties, missing work, disengagement from family or home life). 

- An increase on the PHQ-9 item 9 from baseline 

- A sustained and significant increase in symptoms on the PCL-5 or PHQ-9  (e.g., 10 or more on the PCL-5 or 5 or more on the PHQ-9). 

- Suicidal ideation with high intent, but no plan (change from baseline scores)

- Drop out of study procedures.  

- Any actual or potential breach of confidentiality. 

Reporting of Adverse Events. 

All SAEs that relate to the SHAPE studies will be reported to the TSC in a report received by them prior to each TSC meeting, and during the meeting. It will not be deemed necessary to report SAEs which are not directly related to the SHAPE studies (as determined by the PI), or AEs to the TSC. However, these will be recorded, logged (Appendix B and C) and monitored by the PI and study team. If an AE is reported more than once for a participant, or more than once during the study, it will be treated as an SAE and reported to the TSC. 

Complaints from participants related to the project.

In the event that a complaint is received, the research team led by the PI, will attempt to resolve the issue as much  as is possible within the limits of the study aims. If this is not possible, and the issue remains unacceptable to participants, they will be reminded of the voluntary nature of the study. Formal complaints will be logged and dealt with by the sponsor’s representative (Chair of the Research Ethics Committee at the University of Oxford) in liaison with the PI. Those indicating an AE or SAE will be logged accordingly. 

Enquiring about AEs with study participants. 

Some of the AEs documented in the AEs and SAEs protocol will be assessed through questionnaires and interviews administered as part of the study procedures. If any of these assessments indicate the presence of an SAE or AE, or any SAE or AE is reported outside of routine study procedures, further investigation will be made, and the above procedures will be followed where applicable. 

APPENDIX B

SHAPE Adverse Event (AE) and Serious Adverse Event (SAE) Report Form

Please ensure that Serious Adverse Events are sent to Jennifer Wild (jennifer.wild@psy.ox.ac.uk) within 24 hours.

| Date form completed |  |
| --- | --- |
| Type of report | Initial Report  Follow up Report |
| Participant ID |  |
| Reporting Assessor/Coach Name |  |
| Who is the event in relation to? | Participant                Coach                                               Assessor |
| Is this a SAE ? i.e., Admission to psychiatric hospital; Sectioned under the Mental Health Act; Significant and sustained deterioration of pre-existing mental health condition or symptoms that require immediate intervention that cannot be accommodated within the study protocol; Active suicidal behaviour with a plan; Diagnosis of a new mental health condition; A serious safeguarding issue is revealed (i.e., harm to self or others). |
| Is this an AE ? i.e., One or more aspects of the study procedure induces unacceptable levels of distress for either the participant, or a member of the research team A sustained and significant increase in detrimental behaviours (e.g., behavioural difficulties, missing work, disengagement from family or home life) The emergence of new detrimental behaviours (e.g. self-harm, suicidal ideation, safety seeking behaviours, behavioural difficulties, missing work, disengagement from family or home life). An increase on the PHQ-9 item 9 from baseline A sustained and significant increase in symptoms on the PCL-5 or PHQ-9  (e.g., 10 or more on the PCL-5 or 5 or more on the PHQ-9). Suicidal ideation with high intent, but no plan (change from baseline scores) Drop out of study procedures. Any actual or potential breach of confidentiality. |
| Type of event: | Admission to psychiatric hospital;                               Sectioned under the Mental Health Act;                    Significant and sustained deterioration of pre-existing mental health condition or symptoms that require immediate intervention that cannot be accommodated within the study protocol;                                            Active suicidal behaviour with a plan;                        Diagnosis of a new mental health condition;            A serious safeguarding issue is revealed (i.e., harm to self or others).      One or more aspects of the study procedure induces unacceptable levels of distress for either the participant, or a member of the research team                                                 A sustained and significant increase in detrimental behaviours (e.g., behavioural difficulties, missing work, disengagement from family or home life).     The emergence of new detrimental behaviours (e.g. self-harm, safety seeking behaviours, behavioural difficulties, missing work, disengagement from family or home life).     An increase on the PHQ-9 item 9 from baseline A sustained and significant increase in symptoms on the PCL-5 or PHQ-9  (e.g., 10 or more on the PCL-5 or 5 or more on the PHQ-9).    Suicidal ideation with high intent, but no plan (change from baseline scores)         Voluntary drop out of study procedures.                                                                    Involuntary drop out of study procedures.                                      Any actual or potential breach of confidentiality.      Other, if so, please describe below: |
| Was the event unexpected*? | Yes No |
| *Unexpected is defined as an event that was not previously outlined by the information sheet, consent form, protocol, or Safeguarding protocol. |
|  |

| Reported start date of the adverse event |  |
| --- | --- |
| Date PI (Jennifer Wild) made aware of event |  |
| Participant outcome | Resolved   Resolved with sequelae              Ongoing  Death  (date of death) ________ Unknown |
| Date SAE or AE resolved |  |
| Action taken with participant | Continued with study  Discontinued study |
| Intensity (mild = 1; moderate = 2; severe = 3): |  |
| Frequency of event, if known (i.e. if this type of event has been reported before, how many times for this participant and how many times in the study overall?) |  |
| Brief Narrative (describe the events/conversations occurring immediately before, during and following the adverse event; OR describe the onset, symptoms, diagnosis and treatment of the adverse event.) |

| Causality | In your opinion, how likely do you believe that this event was related to the study? Definitely related   Likely to be related   Possibly related                Unlikely to be related   Definitely not related   Not able to tell |
| --- | --- |
|  | If you believe the SAE/AE to be related to the study, which aspect do you believe it is most likely to be related to? The treatment   The assessments                            The waitlist                   The time burden of the study     Other (please specify)      N/A |
| Notes on this |  |
| Is this a serious event?  (i.e. did the event require immediate implication of risk/safeguarding procedures?) | No Yes If ‘yes’, please give reason: Results in death                                         Is life-threatening                Requires inpatient hospitalisation              Results in persistent or significant disability/incapacity                                                            Other                  If other, please specify: |
| SAE Unique Number (for trial lead to complete): | # |

Contacts for additional information:

| Name of reporting coach/assessor |  |
| --- | --- |
| Phone |  |
| Email |  |
| Research Site (Oxford/remote) |  |
| Participant GP details |  |

APPENDIX C

SHAPE PI Report Form for Serious Adverse Events (SAEs)

| Serious Adverse Event Details: |
| --- |
| Date report received: | Research Site: | Participant ID number: | Participant Initials: | SAE Unique number: |
| _ _ /_ _ /_ _ _ _ |  |  |  |  |

| PI assessment of seriousness: |
| --- |
| Do you agree that the event should be classified as a Serious Adverse Event, as per the information available in the “SHAPE AE and SAE Report Form”? |
| Yes No |
| If no, a review should be held with the reporting coach/assessor and if possible, the participants GP to discuss this. If all agree, a signed and dated follow-up report confirming the amended information should be attached to the original form. Please note, an event can only be downgraded with agreement from both the assessor/coach, designated GP and PI. |

| Assessment of intensity: |
| --- |
| Do you agree with the assessment of intensity, as per the information available in the “SHAPE AE and SAE Report Form”? |
| Yes No |
| If no, please give an alternative assessment and indicate whether the original assessment has been downgraded, or upgraded. |
| Mild Moderate        Severe |
| Downgraded Upgraded |
| If changed, please provide an explanation for why the assessment of intensity has been upgraded/downgraded, as appropriate. |
|  |
| If downgraded, a review should be held with the reporting clinician, their supervisor and the site lead to discuss this. If all agree, a signed and dated follow-up report confirming the amended information should be attached to the original form. Please note, an event can only be downgraded with agreement from both the assessor/coach, designated GP, and PI. |

| Assessment of causality (relationship to participation in the trial): |
| --- |
| Relationship | Description | Reasonable possibility that the SAE may have been caused by the intervention? |
| Unrelated | There is no evidence of any causal relationship with the trial/intervention | No |
| Unlikely | There is little evidence to suggest there is a causal relationship with the trial/intervention (e.g. the event did not occur within a reasonable time after the intervention). There is another reasonable explanation for the event (e.g. the participant’s clinical condition, other concomitant treatment). | No |
| Possible | There is some evidence to suggest a causal relationship with the trial/intervention (e.g. because the event occurs within a reasonable time after the intervention). However, the influence of other factors may have contributed to the event (e.g. the participant’s clinical condition, other concomitant treatments). | Yes |
| Probable | There is evidence to suggest a causal relationship, and the influence of other factors is unlikely. | Yes |
| Definite | There is clear evidence to suggest a causal relationship, and other possible contributing factors can be ruled out. | Yes |
|  |
| Do you agree with the assessment of causality, as per the information available in the “SHAPE AE and SAE Report Form”? |
| Yes. No |
| If no, please give an alternative assessment and indicate whether the original assessment has been downgraded or upgraded. |
| Definitely related*      Probably related*     Possibly related*                                      Unlikely to be related            Definitely unrelated   Not able to tell (* these events are considered to be adverse reactions to participation in the study). |
| Downgraded  Upgraded |
| If changed, please provide an explanation for why the assessment of causality has been upgraded/downgraded, as appropriate. |
|  |
| If downgraded, a review should be held with the reporting clinician, their supervisor and the site lead to discuss this. If all agree, a signed and dated follow-up report confirming the amended information should be attached to the original form. Please note, an event can only be downgraded with agreement from both the assessor/coach, designated GP, and PI. |

| Assessment of relevance to a particular aspect of the study: |
| --- |
| Do you agree with the assessment of relevance, as per the information available in the “SHAPE AE and SAE Report Form”? |
| Yes No |
| If no, please give an alternative assessment. |
| Treatment    Assessments     Time burden        Other |
| If changed, please provide an explanation for this change. |
|  |

| Primary Investigator assessment of expectedness |
| --- |
| Do you agree with the assessment of expectedness, as per the information available in the “SHAPE AE and SAE Report Form”? |
| Yes No |
| If no, please give an alternative assessment. |
| Expected     Unexpected     Unclear* (*some events may have been outlined in the information leaflet, consent form or protocol, but presented as very rare. In this case, please indicate as “unclear”. Occurrences of these events will need to be discussed in detail with the TSC and potentially reported to ethics.) |
| If changed, please provide an explanation for this change. |
|  |
| Please state the date and version of the documents used for reference as part of this assessment: |
| Information leaflet:     Date: _ _ /_ _ /_ _ _ _    Version: ___ Consent/assent form: Date: _ _ /_ _ /_ _ _ _    Version: ___ Protocol:                        Date: _ _ /_ _ /_ _ _ _    Version: ___ |

| Primary Investigator Signoff: |
| --- |
| PI Signature: |  |
| Printed Name: |  |
| Date: | Date: _ _ /_ _ /_ _ _ _ |
